# Multimodal assessment of peripheral perfusion in critically ill patients: a pilot study

**DOI:** 10.1186/s13613-025-01585-2

**Published:** 2025-10-30

**Authors:** Zoé Demailly, Elena Modica, Eva Vitali, Francisco Sousa, Carlotta Dragoni, Irene Sbaraini Zernini, Claudio Annicchiarico, Charles Dehout, Fabienne Tamion, Emmanuel Besnier, Hassane Njimi, Elaine Cavalcante dos Santos, Fabio Silvio Taccone

**Affiliations:** 1 Department of Intensive Care, Erasme Hospital, Brussels University Hospital, Université Libre de Bruxelles, Brussels, 1070 Belgium; 2 Medical Intensive Care Unit, CHU Rouen, Normandie Université, Rouen, 76000 France

**Keywords:** Peripheral perfusion, Microcirculatory perfusion, Peripheral perfusion, Haemodynamic, Critically ill, Intensive care unit

## Abstract

**Background:**

Impaired peripheral perfusion is linked to poor outcomes in critically ill patients, but the relationships among common bedside assessment tools remain unclear. This study aimed to evaluate whether these parameters provide overlapping or complementary prognostic information across ICU subgroups.

**Methods:**

Adult ICU patients with an expected stay ≥ 3 days were included. On day 1, six peripheral perfusion parameters were simultaneously measured: Peripheral Perfusion Index (PPI), Mottling Score (MS), Capillary Refill Time (CRT), central-to-peripheral temperature gradient (ΔT), Skin Blood Flow at basal temperature (SBF_BT_), and forearm tissue oxygenation (rSO₂). The primary outcome was correlation between parameters; secondary outcomes included subgroup consistency and associations with ICU mortality.

**Results:**

Fifty-five patients were included (median age 64; 65.5% male). Circulatory shock (36.4%) was the leading admission cause, followed by acute brain injury (ABI; 29.1%) and acute respiratory failure (ARF; 25.4%). Strong correlations were found between PPI, CRT, SBF_BT_, and ΔT, while rSO₂ showed no significant associations. Correlations were strongest in circulatory shock, weaker in ABI and ARF subgroups. CRT had the highest predictive value for ICU mortality (AUC = 0.75, *p* = 0.007), followed by MS (AUC = 0.72), SBF_BT_, and PPI. ΔT showed limited performance, and rSO₂ was the weakest predictor.

**Conclusions:**

Most bedside peripheral perfusion parameters were strongly interrelated, particularly PPI, SBF_BT_, and ΔT. In contrast, rSO₂ appeared poorly correlated and less predictive. CRT emerged as the most reliable marker of ICU mortality.

**Supplementary Information:**

The online version contains supplementary material available at 10.1186/s13613-025-01585-2.

## Background

Microcirculatory (i.e., peripheral) perfusion is increasingly recognized as a key determinant of outcomes in critically ill patients [1–4]. While traditional resuscitation strategies focus on macrocirculatory targets such as blood pressure and cardiac output [5], studies have shown that restoring these parameters does not guarantee adequate tissue oxygenation [6, 7]. Alterations in the microcirculation are common in critical illness and have been linked to organ failure and poor prognosis [8, 9]. Importantly, microcirculatory dysfunction can occur even in the absence of overt macrocirculatory abnormalities [10, 11]. The loss of coherence between macro- and microcirculation underscores the need for a more integrated hemodynamic approach [12–14], especially as improvements in microcirculatory perfusion have been linked to better organ function and survival [15], highlighting its relevance as a therapeutic target [16].

Currently, a variety of techniques are available to assess microcirculatory perfusion [17, 18]. However, no tool currently exists that can provide an exhaustive and simultaneous assessment of all key microcirculatory parameters, including capillary density, heterogeneity and microvascular flow, vasoreactivity, capillary recruitment, as well as the efficiency of oxygen diffusion and uptake at the tissue level. While some studies focused on clinical evaluation (e.g. mottling score [19] and capillary refill time [20]), others used instrumental assessment such as laser doppler flowmetry [21, 22], plethysmography [23], near-infrared spectroscopy (NIRS) [24, 25] or direct visualization of microvascular flow using handheld video-microscopy [26].. Although inconsistencies between techniques might be viewed as a limitation, they may instead reflect the complex and multifaceted nature of microcirculatory dysfunction. A multimodal monitoring approach could offer a more comprehensive characterization of these alterations, enabling clinicians to target specific abnormalities identified by one or more modalities, rather than relying on a single assessment technique. Interestingly, whether these various tools provide convergent or divergent assessments of peripheral perfusion is poorly studied, as comparative studies are scarce and in most cases only evaluate two techniques at a time. [27–30]. Thus, this pilot study aimed to evaluate the correlation among the most commonly used devices in the Intensive Care Unit (ICU) for assessing peripheral perfusion alterations. It also explored whether these tools yield distinct or complementary prognostic information over time across different subpopulations of critically ill patients – specifically, patients with circulatory shock, who may serve as a reference group given the well-established presence of microcirculatory alterations [4, 7, 8, 12], and patients with acute brain injury (ABI) and acute respiratory failure (ARF), populations in whom peripheral perfusion remains largely unexplored.

## Methods

### Study design and setting

We conducted a prospective, monocentric study cohort in a general 34-bed ICU of the Hôpital Universitaire de Bruxelles (HUB), Belgium, from July 2024 to February 2025. The study was approved by the Ethics Committee (protocol number P2024/776), and informed consent was obtained from all patients or their next of kin.

### Study population

We included adult patients admitted to the ICU without significant hemodynamic variations (e.g. mean arterial pressure, MAP ≥ 65 mmHg and, if on vasopressors, no change in drug regimen for at least 2 h) following initial resuscitation, and whose expected length of stay (LOS) in ICU was at least 3 days, based on the opinion of the treating medical team. Exclusion criteria were pregnancy, peripheral vascular disease, ineligibility for skin perfusion assessment (e.g. amputation of fingers or legs; presence of any skin lesion at the site of measurement), circulatory assist devices (alteration in peripheral pulsatility) or active renal replacement therapy (fluid removal during therapy can acutely alter peripheral perfusion parameters [21]). Subgroups were defined as follows: circulatory shock was identified by the need for vasopressor support after fluid resuscitation optimization, in combination with a lactate level ≥ 2 mmol/L; ABI was defined as acute and severe brain insult resulting from primary stroke, either haemorrhagic or ischaemic, or from traumatic brain injury ; ARF was defined as the inability of the respiratory system to maintain adequate gas exchange, requiring non-invasive ventilation, or invasive mechanical ventilation.

### Data collection and monitoring

General characteristics of the patients were recorded, including demographics, reason for admission, and severity of illness assessed by the Sequential Organ Failure Assessment (SOFA) score [31] on days 1 to 3, as well as the Simplified Acute Physiologic Score II (SAPS II) [32] on day 1. We also recorded survival status at ICU discharge. The recorded parameters included general hemodynamic variables, e.g. mean arterial pressure (MAP), heart rate (HR), cardiac index (CI) whenever available, as well as vasoactive drug support using the maximal Vasoactive Inotropic Score (VIS) [33], defined as the highest score recorded on the day of measurement, quantifying the maximal load of vasoactive and inotropic agents administered, thus reflecting the intensity of pharmacological circulatory support. When feasible, arterial and central venous or mixed venous blood samples were collected simultaneously at each time point to measure blood gases (in particular, the veno-arterial difference in partial pressure of CO_2_ (gapPCO_2_), the arteriovenous oxygen difference in (DAV-O_2_)) and lactate levels.

### Peripheral perfusion assessment

Various assessments of the peripheral perfusion were performed on day 1, day 2 (24 h ± 3 h after day 1 measurements), and day 3 (48 h ± 3 h after day 1 measurements). All measurements were conducted under standardized ICU conditions, including controlled ambient temperature and optimized lighting, to ensure accurate and reproducible bedside peripheral perfusion assessments. Peripheral perfusion index (PPI), defined as the difference between the pulsatile and non-pulsatile portion of pulse wave, was measured using a fingertip pulse oximeter (Massimo Corporation, Irvine, CA, USA). Mottling score (MS) was based on visual quantification of the extension of the mottled area on the legs (score 0 = no mottling; score 1 = a small area of mottling, the size of a coin, located in the center of the knee; score 2 = an area of mottling that does not extend beyond the upper edge of the knee cap; score 3 = an area of mottling that does not extend beyond the middle of the thigh; score 4 = an area of mottling that does not extend beyond the groin crease; score 5 = an area of extremely severe mottling that extends beyond the groin crease) [19]. Capillary refill time (CRT) was measured once at each timepoint, according to Hernandez et al. method [20], by firmly pressing a glass slide on the ventral surface of the index finger until the skin turns white, maintaining the pressure for 10 s, and then releasing. The time to return to normal skin color was measured with a chronometer upon release; a refill time greater than 3 s was considered “abnormal”. Central-to-peripheral temperature gradient (ΔT) was determined by subtracting the skin temperature (measured using a laser doppler probe placed on the index finger) from the core temperature (measured with a digital thermometer placed under the armpit and corrected by adding 0.27 °C [34]). Skin blood flow at basal temperature (SBF_BT_) measurements were performed on the palmar surface of the tip of the index finger at basal skin temperature using a skin laser doppler device (PeriFlux System 5000, Perimed, Jarfalla, Sweden), with a small thermostatic probe (Reference number 457, Perimed). Data were recorded continuously for 3 min for subsequent offline analysis using the PeriSoft 2.5.5 software (Perimed), and the value retained for analysis was the average of the last 30 s of the recording. Regional tissue oxygenation (rSO_2_) was measured using near-infrared spectroscopy (NIRS) of the O3™ device (Masimo Corporation, Irvine, CA, USA) on the ventral side of the forearm. Measurements were performed on the side contralateral to the radial arterial catheter, when present.

### Study outcomes

The primary outcome was to evaluate the correlation between peripheral perfusion and oxygenation parameters on the first day (D1) of ICU admission, including continuous variables such as PPI, CRT, ΔT, SBF_BT_, and rSO2. The mottling score (MS), as a categorical variable, was not included in the correlation matrix but was analyzed separately as a secondary outcome. Secondary outcomes also included assessing these correlations separately in the circulatory shock and ABI / ARF groups patients, and whether these variables were associated with ICU mortality. Sensitivity analyses were performed to evaluate the concordance of changes in these parameters over the first three days of ICU stay (D1 to D3).

### Statistical analysis

Continuous variables are presented as mean ± standard deviation (SD) or median [interquartile range], while categorical variables are reported as counts and percentages (n, %). Group comparisons were performed using the Man-Whitney test for continuous variables and either Pearson’s Chi-square test or Fisher’s exact test for categorical variables, as appropriate. Microcirculatory variables were tested for normality using the Shapiro-Wilk test. Since almost data did not follow a normal distribution, associations between the variables were assessed using Spearman’s rank correlation coefficient (ρ), which does not assume normality. A correlation matrix was generated to evaluate the strength and direction of relationships among the continuous variables. The significance of each correlation was determined using a two-tailed p-value, with adjustments for multiple comparisons using the Bonferroni correction. Results are presented as correlation coefficients (ρ) with their corresponding 95% confidence intervals (CIs) and ρ values interpreted as follows: 0-0.19, very weak ; 0.20–0.39, weak ; 0.40–0.59, moderate ; 0.60–0.79, strong ; and 0.80-1.00, very strong correlation.

Receiver Operating Characteristic (ROC) curve analysis was performed to evaluate the diagnostic performance of microcirculatory parameters in predicting ICU mortality in the overall population. The area under the curve (AUC) was calculated for each parameter, along with corresponding 95% confidence intervals (CI) and p-values. The ROC curves were compared in a paired-sample setting [35], where multiple test values were obtained from the same subjects in relation to a common state variable. Dynamics of microcirculatory parameters correlations over time were assessed using repeated measures correlation statistical technique for determining the common within-individual association for paired measures assessed on two or more occasions for multiple individuals. Results are presented as correlation coefficients (ρ) with their corresponding 95% CIs. All tests were two-sided, and statistical significance was set at *p* < 0.05. Statistical analyses were performed using GraphPad PRISM (version 10, GraphPad Software, San Diego, CA, USA) and Stata, version 18.5 for Windows (StataCorp LLC).

## Results

### Population characteristics

**Fig. 1:**
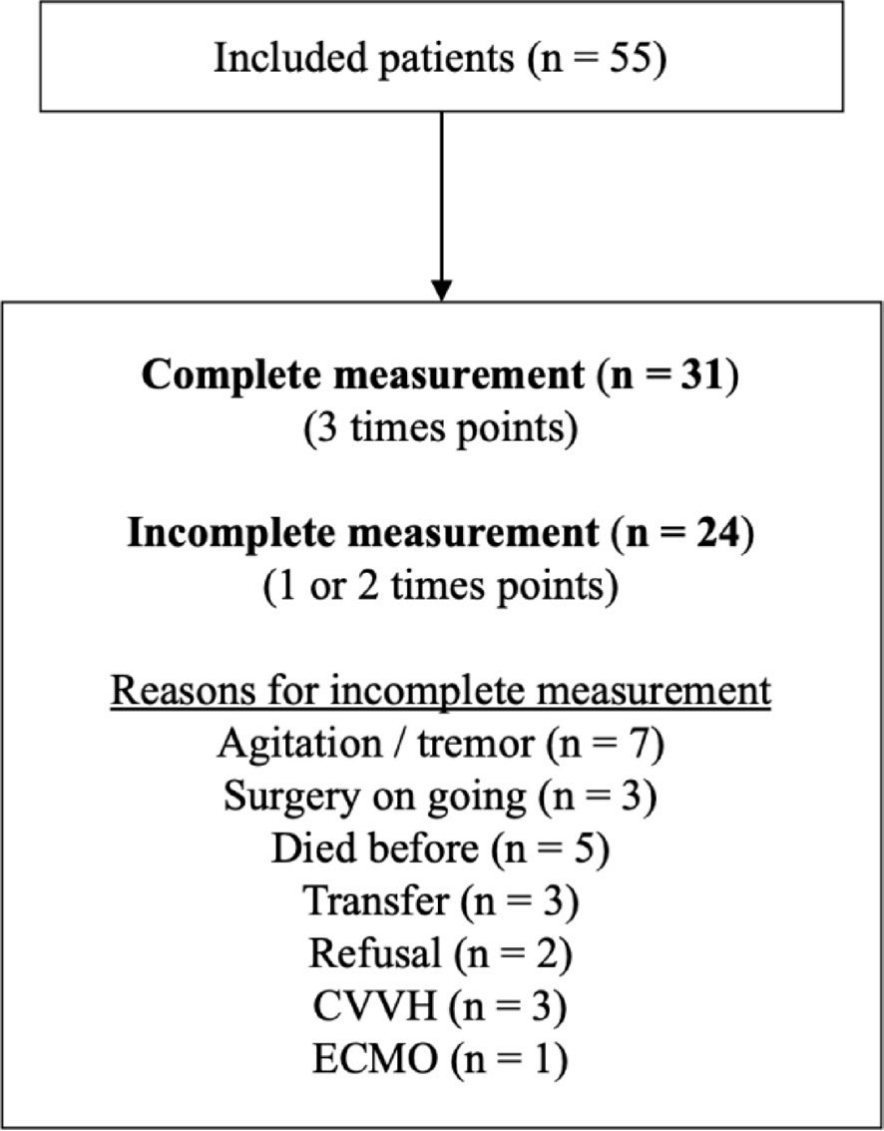
Patients’ inclusion and measurement completion. This diagram illustrates the inclusion process, the number of patients who received one, two, or three measurements, and the reasons for incomplete data collection. CVVH: Continuous Veno-Venous Hemofiltration; ECMO: Extracorporeal membrane oxygenation

A total of 55 patients were included. Of these, 31 (56%) received all three measurements. The inclusion follow-up and details of the reasons for incomplete measurements are provided in Fig. 1. The primary reason for admission was circulatory shock (*n* = 20, 36.4%), followed by neurological failure (*n* = 16, 29.1%) and respiratory failure (*n* = 14, 25.4%). The median LOS in the ICU was 5 [4–16] days. Major comorbidities, severity scores, and other key characteristics of the study population are presented in Table 1.

**Table 1:** Baseline patient characteristics and outcomes

	Population (*n* = 55)
Age, years	64 [55–73]
Male gender, n (%)	36 (65.5)
*Comorbidities*	
Obesity*	14 (25.5)
Arterial hypertension	27 (49.1)
Diabetes	19 (34.5)
Ischemic cardiopathy	5 (9.1)
Chronic heart failure	7 (12.7)
Chronic atrial fibrillation	6 (10.9)
Chronic Renal Failure	8 (14.5)
COPD	7 (12.7)
Cancer	10 (18.2)
Cirrhosis	6 (10.9)
Immunodeficiency	3 (5.5)
Alcohol	13 (23.6)
Smoking	17 (30.9)
*Reason for admission*	
Circulatory shock	20 (36.4)
Acute brain injury	16 (29.1)
Acute respiratory failure	14 (25.4)
Acute hepatic failure	4 (7.3)
Severe metabolic disorders	1 (1.8)
*SOFA scores*	
Day 1	6 [4–10]
Day 2	5 [3–9]
Day 3	5 [ 2–8]
SAPS II	44.7 ± 17.9
*LOS*	
ICU	6 [3–15]
Hospital	23 [10–46]
ICU mortality	14 (25.5)

Data are presented as numbers (percentages). mean ± standard deviation, or median [interquartile range] according to the distribution. *BMI : body mass index ; COPD*: Chronic Obstructive Pulmonary Disease ; ICU : intensive care unit ; SAPS II : Simplified Acute Physiology Score ; SOFA Sepsis-related Organ Failure Assessment. * Body Mass Index > 30 kg/m^2^

On D1, the median values for key macrocirculatory and biological parameters in this overall population were as follows: HR was 87 bpm (69–98), MAP was 78 mmHg (70–89) and maximal VIS was 5.0 (0.0–25.3). Lactate level was 1.7 mmol/L (1.0–2.8). The PCO₂ gap was 6.0 mmHg (4.8–9.3), while the arteriovenous oxygen difference (DAVO₂) had a median of 3.45 (2.91–4.64) mL/100 mL. In the circulatory shock and ABI groups, the maximal VIS were 15.5 (4.8–58) and 0 (0–14.2), respectively.

**Table 2:** Macrocirculatory and biological parameters measured on day 1 (D1) in the overall population and sub-groups

Macrocirculatory and biological parameters on D1	All (*n* = 55)	Circulatory shock (*n* = 20)	ABI (*n* = 16)	ARF (*n* = 14)
Heart Rate (bpm)	87 (69–98)	90.5 (71–107)	72 (64–90)	85 (70–100)
MAP (mmHg)	78 (70–89)	71 (68–80)	92 (79–100)	72 (69–81)
VISmax	5.0 (0.0–25.3)	15.5 (4.8–58.0)	0.0 (0.0–14.2)	6.4 (0.0–23.4)
Lactate (mmol/L)	1.7 (1.0–2.8)	2.4 (1.2–7.8)	1.1 (0.8–1.9)	1.6 (1.2–2.3)
PCO₂ gap (mmHg)	6.0 (4.8–9.3)	7.0 (5.0–10.0)	4.0 (2.0–6.5)	5.0 (5.0–11.0)
AV O₂ diff. (mL/100 mL)	3.45 (2.91–4.64)	3.61 (3.13–4.91)	3.11 (2.43–4.38)	3.33 (2.88–4.97)

ARF: Acute Respiratory Failure; ABI: Acute Brain Injury; AV O₂ diff.: Arteriovenous O₂ difference; MAP: Mean Arterial Pressure; VISmax: Maximal Vasopressor Index Score

### Primary outcome

**Fig. 2:**
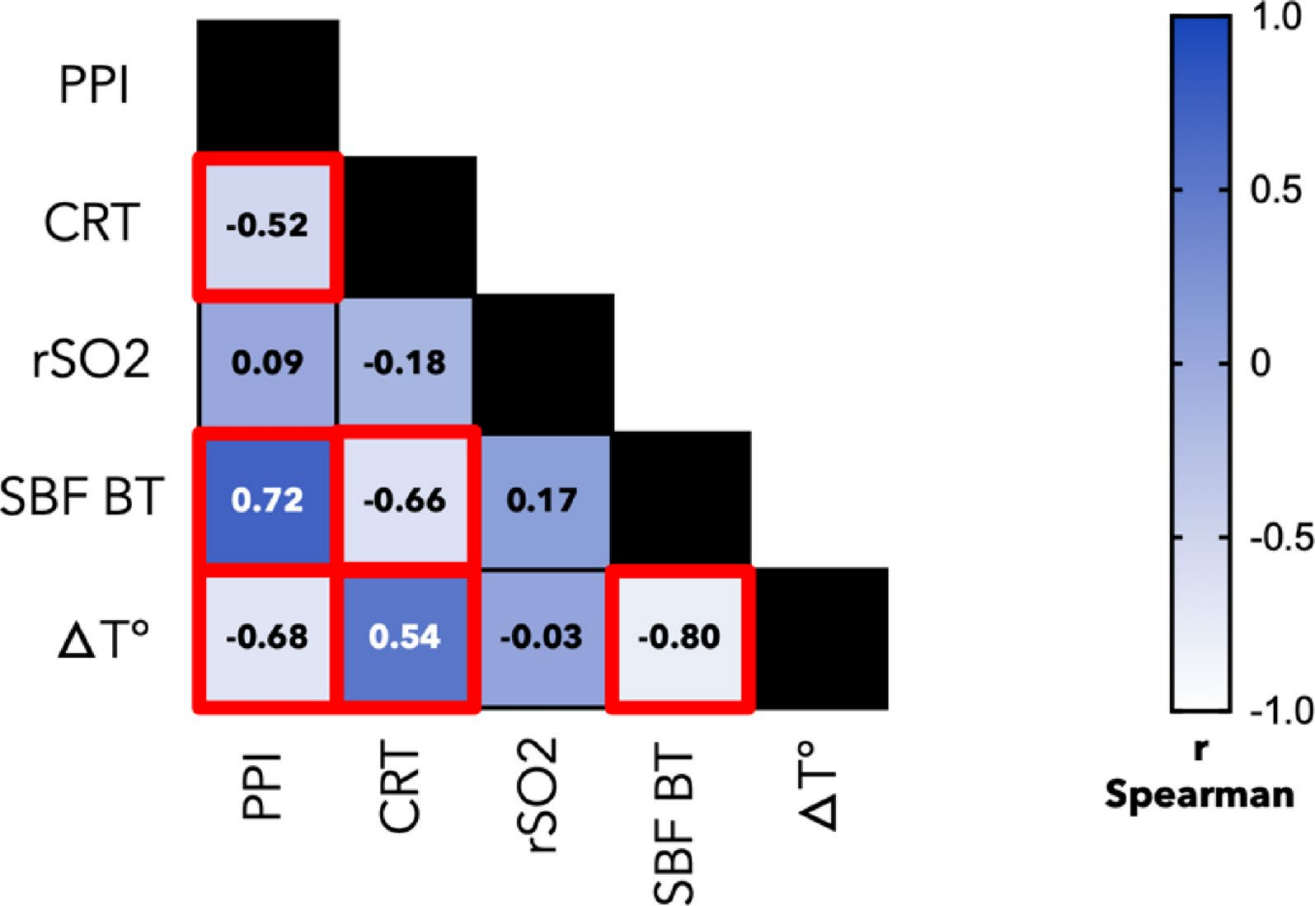
Correlation matrix of the studied parameters in the overall population on day 1. This matrix displays the Spearman correlation coefficients (ρ) between peripheral perfusion index, capillary refill time, regional tissue oxygenation, skin blood flow at basal temperature, and the central-to-peripheral temperature gradient. Significant correlations (*p* < 0.005) are highlighted in red. CRT: capillary refill time; rSO2: regional tissue oxygenation; PPI: peripheral perfusion index; SBF_BT_: skin blood flow at basal temperature; ΔT°: central-to-peripheral temperature gradient

All day 1 peripheral perfusion parameters were collected at a median time of 18.5 hours (IQR: 12–23 h) after ICU admission. PPI showed a strong positive correlation with SBF_BT_ (ρ = 0.72, *p* < 0.001) and a strong negative correlation with ΔT (ρ = -0.68, *p* < 0.001). CRT was strongly negatively correlated with SBF_BT_ (ρ = -0.66, *p* < 0.0001) and positively correlated with ΔT (ρ = 0.54, *p* < 0.001). Additionally, CRT and PPI showed a moderate negative correlation (ρ = -0.52, *p* < 0.001); SBF_BT_ exhibited a strong negative correlation with ΔT (ρ = -0.80, *p* < 0.001) and rSO_2_ did not show significant correlations with the other variables. The correlations between the different parameters are illustrated in Fig. 2.

### Secondary outcomes

**Fig. 3:**
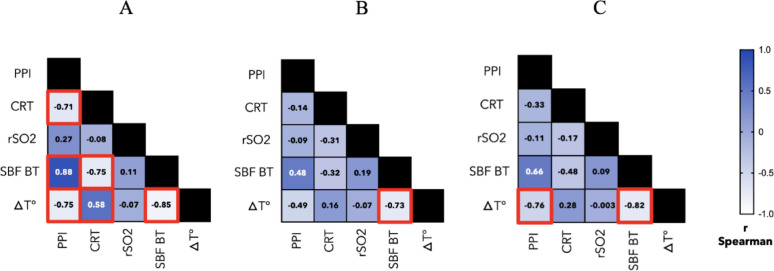
Subgroups correlation matrix of the studied parameters on day 1. This matrix displays the Spearman correlation coefficients (ρ) between peripheral perfusion index, capillary refill time, regional tissue oxygenation, skin blood flow at basal temperature, and the central-to-peripheral temperature gradient in circulatory shock group (**A**), acute brain injury group (**B**) or acute respiratory failure group (**C**). Significant correlations (*p* < 0.005) are highlighted in red. CRT: capillary refill time; rSO2: regional tissue oxygenation; PPI: peripheral perfusion index; SBF_BT_: skin blood flow at basal temperature; ΔT: central-to-peripheral temperature gradient

In the subgroup analyses, the same associations were observed and strengthened in patients with acute circulatory shock (Fig. 3, A), whereas in patients with ABI, only the association between SBF_BT_ and ΔT was maintained (Fig. 3, B), and in patients with ARF, associations were maintained between ΔT and PPI, and between ΔT and SBF_BT_ (Fig. 3, C).

Peripheral perfusion parameters on D1 differed between survivors and non-survivors (Table 2). Among the 14 non-survivors, 9 out of 20 (45%) were admitted for circulatory shock, 4 out 16 (25%) for ABI, and 1 out of 14 (7,1%) for ARF; 9 had care limitations, and 5 died from refractory multiple organ failure without such limitations. In non-survivors, PPI and SBT_BT_ were significantly lower than in survivors (0.95 [0.46–2.14] vs. 2.64 [1.21–4.67], *p* = 0.04 ; 37.8 [11.9–160.3] PU vs. 126.5 [56.5–262.8] PU, *p* = 0.03, respectively), MS was higher (1 [0–2] vs. 0 [0–0], *p* = 0.003) and CRT was longer in non-survivors (2.52 [1.80–4.09] s vs. 1.41[1.00–2.67] s, *p* = 0.006) than in survivors. No significant difference was observed for ΔT and rSO_2_ values between the two groups (*p* = 0.09 and *p* = 0.47, respectively) (Table 3).

**Table 3:** Microcirculatory parameters at day 1 according to vital status at ICU discharge

Parameters	Total (*n* = 55)	Survivors (*n* = 41)	Non-survivors (*n* = 14)	*p*-value
Temperature gradient (°C)	6.8 (3.4–10.4)	6.3 (3.1–10.1)	8.1 (6.3–10.9)	0.09
Perfused Peripheral Index	1.90 (0.81–4.39)	2.64 (1.21–4.67)	0.95 (0.46–2.14)	0.04
Mottling score Score ≥ 1	0 (0–1) 16 (30%)	0 (0–0) 9 (22%)	1 (0–2) 7 (50%)	0.003
Capillary refill time (sec)	1.90 (1.12–2.74)	1.41 (1.00–2.67)	2.52 (1.80–4.09)	0.006
rSO_2_ (%)	64.0 (61.0–70.5)	64.5 (62.0–69.8)	62.5 (51.0–72.8)	0.47
Skin Blood Flow (PU)	88.5 (21.1–255.3)	126.5 (56.5–262.8)	37.8 (11.9–160.3)	0.03

Data are presented as n (%), or median [interquartile range]

**Fig. 4:**
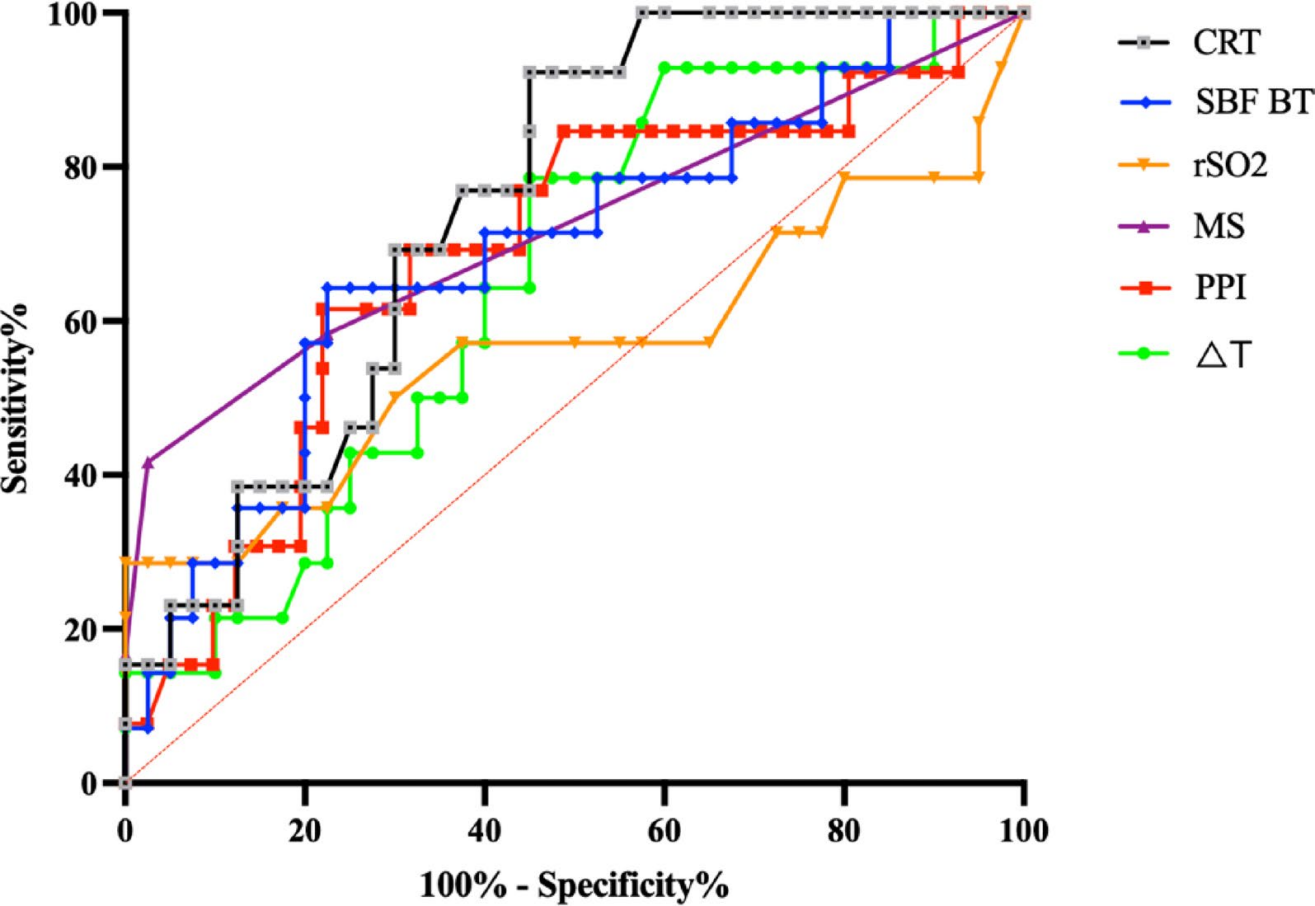
Prediction of ICU mortality by baseline microcirculatory parameters in the overall population. Each ROC curve corresponds to a specific microcirculatory parameter, evaluated in terms of sensitivity and specificity. CRT: capillary refill time;MS: mottling score; rSO2: regional tissue oxygenation; PPI: peripheral perfusion index; S_BT_BT: skin blood flow at basal temperature; ΔT: central-to-peripheral temperature gradient

Twenty percent (11/55) of patients had an abnormal CRT (> 3 s), including 40% (8/20) in the circulatory shock subgroup, 18.8% (3/16) in the ABI subgroup and 0% in the ARF group. The diagnostic accuracy of microcirculatory parameters on D1 to predict ICU mortality in the overall population was assessed using ROC curve analysis (Fig. 4). On D1, CRT demonstrated the highest predictive value for ICU mortality, with an AUC of 0.75 (95% CI: 0.62–0.88, *p* = 0.007). The MS also showed good discriminative ability, with an AUC of 0.72 (95% CI: 0.53–0.91, *p* = 0.021). SBF_BT_ and PPI were significant predictors as well, with AUCs of 0.69 (95% CI: 0.53–0.86, *p* = 0.035) and 0.69 (95% CI: 0.51–0.85, *p* = 0.042) respectively, while ΔT and rSO_2_ were not.

Comprehensive results, including AUCs and 95% confidence intervals for all studied parameters in the overall population are available in Supplementary Table 1. In the circulatory shock group, none of the parameters demonstrated significant discriminatory ability. In patients with ABI, CRT showed the highest predictive value, with an AUC of 0.92 (95% CI: 0.75–1.00, *p* = 0.03). Other microcirculatory parameters showed lower and non-significant predictive performance (Table 4).

**Table 4:** Summary of diagnostic performance of receiver operating characteristic curves for predicting the ICU mortality in patients with circulatory failure and acute brain injury

	CRT	SBT BT	rSO2	MS	PPI	Δ T
*Circulatory shock subgroup*						
AUC	0.52	0.60	0.53	0.60	0.58	0.62
95% CI	0.25–0.79	0.35–0.86	0.25–0.81	0.33–0.87	0.31–0.84	0.37–0.88
p value	0.849	0.425	0.820	0.457	0.569	0.362
*Acute brain injury subgroup*						
AUC	0.92	0.73	0.55	0.81	0.75	0.58
95% CI	0.75–1	0.46–1	0.15–0.97	0.44–1	0.47–1	0.26–0.90
p value	0.03	0.182	0.744	0.112	0.194	0.628

The AUC (area under the curve), 95% confidence intervals (CI), and p-values are presented for each microcirculatory parameter. The parameters used include Capillary Refill Time (CRT), Skin Blow flow at basal temperature (SBF_BT_), regional tissue oxygenation (rSO2), Mottling Score (MS), Perfused Pulsatility Index (PPI) and the central-to-core gradient temperature (Δ T)

### Dynamics of microcirculatory parameters correlations over time (D1 to D3)

In the overall population, statistically significant correlations were observed between several microcirculatory parameters over time (Supplementary Table 2). The strongest correlation was found between SBF_BT_ and ΔT ( ρ = -0.67, 95% CI: -0.78 to -0.51, *p* < 0.001). PPI correlated positively with SBF_BT_ (ρ = 0.29, *p* = 0.02) and negatively with ΔT (ρ = -0.47, 95% CI: -0.64 to -0.26, *p* < 0.001). CRT was positively correlated with ΔT (ρ = 0.33, *p* = 0.01) and negatively with SBF_BT_ (ρ = -0.25, *p* = 0.04). No significant correlations were found involving rSO_2_.

## Discussion

Our study provide an initial detailed mapping of the peripheral perfusion in critically ill patients using several simple, non-invasive tools commonly used or applicable at the patient’s bedside. Our results revealed strong correlations between several microcirculatory parameters in the overall population and in the circulatory shock subgroup, particularly among PPI, SBF_BT_, and ΔT. In contrast, rSO₂ showed no significant association with the other parameters, suggesting that it may reflect different – potentially complementary – physiological aspects of tissue perfusion. These correlations were not observed in the ABI subgroup, highlighting potential pathophysiological differences in this population.

In the overall population, PPI was strongly positively correlated with SBT_BT_ and negatively with ΔT, reflecting consistent physiological relationships. Indeed, both the PPI and SBF_BT_ provide insight into the pulsatile and non-pulsatile components of distal peripheral blood flow without distinguishing between arterioles, venules or capillaries [36]. It is also consistent that the thermal gradient, which is influenced by vasoconstriction and increases when vessel caliber and peripheral blood flow decrease [36], would be inversely correlated with these two parameters. CRT, which reflects microvascular reactivity and increases when this reactivity is impaired (delayed return of blood to the capillaries), was also well correlated with SBT_BT_ (negative correlation) and with ΔT (positive correlation), while being inversely associated with the PPI. Regional SO_2_, on the other hand, showed no significant correlation with the other variables studied, questioning its relevance in this population or its usefulness as a direct reflection of peripheral perfusion. However, this result appears to differ from that of Lima et al. [37], who reported a significant difference in NIRS values between two groups of patients with or without impaired peripheral circulation, defined in their study by a temperature gradient greater than 4 °C and a prolonged CRT exceeding 4.5 s. This discrepancy may be related to the site of rSO_2_ measurement, which was the thenar eminence in their study, whereas it was the forearm in ours.

In subgroup analyses, the correlations observed were enhanced in patients with acute circulatory shock, suggesting greater sensitivity of these parameters in this context. In contrast, in patients with ABI only the correlation between SBT_BT_ and CRT was maintained, while in ARF subgroup correlation were maintained between ΔT and PPI, and between ΔT and SBF_BT_, likely reflecting a distinct regulation of the peripheral perfusion in these subgroups. The observed loss of coherence between peripheral perfusion parameters in patients with ABI may reflect an early alteration of systemic microcirculatory autoregulation, as previously suggested in preclinical models. Two experimental studies reported both impaired endothelium-dependent vasodilation [38] and dysregulated microcirculatory blood flow, characterized by an overshoot phenomenon following cerebral injury [39]. These findings support the hypothesis that systemic microcirculatory disturbances occur in ABI and may be dissociated from global hemodynamics. In this context, the inclusion of the ABI subgroup in our study was intended to explore whether bedside peripheral perfusion markers could provide additional prognostic or pathophysiological insights in this specific population.

Peripheral perfusion parameters on admission differed between survivors and non-survivors, with significantly lower PPI and SBT_BT_ values, prolonged CRT, and a higher MS in non-survivors. Two other studies investigated the association between various early-measured microcirculatory parameters and patient survival in the ICU. In the first study [40], only CRT was able to provide prognostic information distinguishing survivors from non-survivors, whereas in the second [30], both CRT and SBT_BT_ demonstrated this capability. Our findings are consistent with these results, while enabling the assessment of a broader range of microcirculatory parameters. Among all the parameters evaluated in our study, CRT had the best predictive value for ICU mortality (AUC = 0.75), followed by MS, SBT_BT_ and PPI. However, this predictive performance was lost in the circulatory shock subgroup, likely due to greater heterogeneity in CRT values despite apparent hemodynamic optimization. This variability may reflect the coexistence of multiple shock etiologies, individual patient factors affecting peripheral perfusion, and a dissociation between macrohemodynamic stabilization and microcirculatory recovery, as previously described [41]. The predictive value of CRT was preserved in the acute brain injury subgroup. Conversely, rSO_2_ was unable to distinguish between survivors and non-survivors, preventing any conclusion regarding its prognostic value in the ICU when used as a peripheral perfusion indicator, a topic that has already been debated in the literature [42, 43].

Longitudinal analyses showed that some correlations remained significant over time, especially between SBT_BT_ and ΔT (strong negative correlation), suggesting that either of these two parameters could be used for microcirculatory monitoring over time, depending on their availability in the clinical setting. PPI and CRT also maintained significant correlations with these two parameters, while rSO_2_ showed no significant correlation with the other parameters over time.

Our study presents several limitations. Firstly, its small sample size and monocentric design inherently limit the statistical power and generalizability of the findings. This limitation is particularly relevant in subgroup analyses, such as in patients with circulatory shock, where the lack of significant associations with ICU mortality may reflect insufficient statistical power rather than a true absence of relationship. Additionally, the wide variability in peripheral perfusion parameters observed within this group, despite hemodynamic optimization, may have further influenced their predictive performance. Nevertheless, the study was designed as an exploratory, hypothesis-generating work to assess the feasibility and potential relevance of multimodal peripheral perfusion assessment in a mixed ICU population. Secondly, our daily monitoring approach may have missed short-term fluctuations in tissue perfusion, especially in patients with circulatory shock. However, the study was pragmatically designed to capture a representative snapshot upon ICU admission, consistent with previous studies showing the prognostic value of first-day perfusion assessments [11, 12]. Futhermore, seven patients ultimately had a LOS shorter than expected for various reasons related to the observational nature of the study, which limited the completeness of follow-up data and may have reduced the statistical power of longitudinal analyses. It is also possible that our findings regarding rSO₂ were influenced by the type of sensor used in our study, which had to be placed on the forearm rather than on the thenar eminence, closer to the site of the other measurements. Moreover, the lack of dynamic assessment, such as a vascular occlusion test, may have further limited the ability of rSO₂ measurements to reflect microcirculatory function or correlate with other perfusion indices. Also, it is important to acknowledge that severe circulatory failure may affect the reliability of some of these measurements – such as PPI and SBF – due to signal instability, which represents an intrinsic limitation of bedside peripheral perfusion monitoring. Then, the potential influence of skin pigmentation on clinical assessment tools such as CRT and mottling score cannot be excluded, although standardized conditions inherent to the ICU environment (e.g., optimized lighting and temperature) were maintained. Also, it is important to note that core temperature was estimated from axillary measurements as per routine practice in our center, but we applied a correction to ensure comparability with published data. Furthermore, we were unable to compare these peripheral perfusion measurements to the current gold standard for microcirculatory assessment – sublingual microcirculation imaging using sidestream dark field or incident dark field technologies – which could have further strengthened our findings and helped identify potential surrogates for this time-consuming and technically demanding bedside evaluation in critically ill patients.

## Conclusions

In this study, peripheral perfusion parameters correlated differently depending on the underlying condition, with stronger associations in patients with circulatory shock. CRT emerged as the most reliable marker of ICU mortality. Based on our data, we cannot suggest that peripheral perfusion indices are interchangeable and no single tool appears to be able to provide a comprehensive assessment of microcirculatory function in critically illness. Bedside interpretation of all these tools should be adapted to the patient’s clinical phenotype, disease severity, and predominant circulatory derangement to optimize their diagnostic and prognostic value.

## Supplementary Information

Additional file 1.

Additional file 2.

## Data Availability

The datasets supporting the conclusions of this article are included within the article, and its additional files.

## Abbreviations

AUC: Area under the curve
CI: Confidence intervals
CRT: Capillary refill time
LOS: Length of stay
MS: Mottling score
NIRS: Near-infrared spectroscopy
PPI: Peripheral perfusion index
ROC: Receiver operating characteristic
rSO2: Regional tissue oxygenation
SAPS II: Simplified acute physiologic score II
SBF_BT_: Skin blood flow at basal temperature
SOFA: Sequential organ failure assessment
ΔT: Central-to-peripheral temperature gradient
