# Supplementary material for: Multimodal assessment of peripheral perfusion in critically ill patients: a pilot study
Source: Ann Intensive Care. 2025 Oct 30;15:176. doi: 10.1186/s13613-025-01585-2 (PMC12575891; doi:10.1186/s13613-025-01585-2)
Supplement: Supplementary file 1 — Additional file 1. [file 13613_2025_1585_MOESM1_ESM.docx]

|  | **CRT** | **SBT BT** | **rSO2** | **MS** | **PPI** | **Δ T** |
| --- | --- | --- | --- | --- | --- | --- |
| **AUC** | 0.75 | 0.69 | 0.57 | 0.72 | 0.69 | 0.66 |
| **95% CI** | 0.62 - 0.88 | 0.53 - 0.86 | 0.36 - 0.77 | 0.53 - 0.91 | 0.51 - 0.85 | 0.50 - 0.81 |
| **p value** | 0.007 | 0.035 | 0.465 | 0.021 | 0.042 | 0.084 |

**Supplementary Table 1.** **Summary of diagnostic performance of ROC curves for predicting the ICU mortality in the overall population.** The AUC (area under the curve), 95% confidence intervals (CI), and p-values are presented for each microcirculatory parameter. The parameters used include Capillary Refill Time (CRT), Skin Blow flow at basal temperature (SBF BT), regional tissue oxygenation (rSO2), Mottling Score (MS), Perfused Pulsatility Index (PPI) and the central-to-core gradient temperature (Δ T).
