# Supplementary material for: Multimodal assessment of peripheral perfusion in critically ill patients: a pilot study
Source: Ann Intensive Care. 2025 Oct 30;15:176. doi: 10.1186/s13613-025-01585-2 (PMC12575891; doi:10.1186/s13613-025-01585-2)
Supplement: Supplementary file 2 — Additional file 2. [file 13613_2025_1585_MOESM2_ESM.docx]

|  |  | **PPI** | | **CRT** | **rSO_2_** | **SBTBT** | **△T** |
| --- | --- | --- | --- | --- | --- | --- | --- |
| **PPI** | ρ |  | | -0.22 | 0.03 | 0.29 | -0.47 |
|  | *Lower 95%CI* |  | | -0.43 | -0.22 | 0.05 | -0.64 |
|  | *Upper 95%CI* |  | | 0.03 | 0.27 | 0.49 | -0.26 |
|  | *p-value* |  | | 0.08 | 0.84 | **0.02** | **<0.001** |
| **CRT** | ρ |  | |  | -0.15 | -0.25 | 0.33 |
|  | *Lower 95%CI* |  | |  | -0.39 | -0.46 | 0.10 |
|  | *Upper 95%CI* |  | |  | 0.10 | -0.01 | 0.53 |
|  | *p-value* |  | |  | 0.23 | **0.04** | **0.01** |
| **rSO_2_** | ρ |  | |  |  | 0.19 | -0.03 |
|  | *Lower 95%CI* |  | |  |  | -0.05 | -0.27 |
|  | *Upper 95%CI* |  |  |  |  | 0.42 | 0.21 |
|  | *p-value* |  |  |  |  | 0.11 | 0.80 |
| **SBTBT** | ρ |  |  |  |  |  | -0.67 |
|  | *Lower 95%CI* |  |  |  |  |  | -0.78 |
|  | *Upper 95%CI* |  |  |  |  |  | -0.51 |
|  | *p-value* |  |  |  |  |  | **<0.001** |
| **△T** | ρ |  |  |  |  |  |  |
|  | *Lower 95%CI* |  |  |  |  |  |  |
|  | *Upper 95%CI* |  |  |  |  |  |  |
|  | *p-value* |  |  |  |  |  |  |

**Supplementary Table 2.** **Spearman correlation coefficients (ρ), 95% confidence intervals (CI), and p-values for pairwise correlations between microcirculatory parameters over time.** *CRT: capillary refill time; rSO_2_: regional tissue oxygenation; PPI: peripheral perfusion index; SBTBT: skin blood flow at basal temperature; ΔT: central-to-peripheral temperature gradient.*
